# Serum cytokine pattern in young children with screening detected coeliac disease

**DOI:** 10.1111/cei.12454

**Published:** 2015-01-02

**Authors:** S Björck, S R Lindehammer, M Fex, D Agardh

**Affiliations:** * Department of Pediatrics, Skåne University HospitalMalmö, Sweden; † Unit of Diabetes and Celiac Disease, Department of Clinical Sciences, Lund UniversityMalmö, Sweden

**Keywords:** coeliac disease, children, cytokines, HLA, tissue transglutaminase antibody

## Abstract

Coeliac disease is an autoimmune disease characterized by inflammation localized to the small bowel, but less is known about systemic signs of inflammation. The aim was to measure cytokines of the T helper 1 (Th1) and T helper 2 (Th2) cell patterns in children with screening-detected coeliac disease before and after treatment with a gluten-free diet. Serum samples selected before and after the start of a gluten-free diet from 26 3-year-old children diagnosed with biopsy-proven coeliac disease and from 52 matched controls were assayed in an multiplex enzyme-linked immunosorbent assay (ELISA) for the 10 cytokines: interferon (IFN)-γ, interleukin (IL)-1β, IL-2, IL-4, IL-5, IL-8, IL-10, IL-12p70, IL-13 and tumour necrosis factor (TNF)-α. Among Th1 cytokines, IFN-γ and IL-12p70 were elevated significantly in children with coeliac disease compared to controls (*P* < 0·001 and *P* = 0·001, respectively). Similar findings were demonstrated for the Th2 cytokines IL-5 (*P* < 0·001), IL-10 (*P* = 0·001) and IL-13 (*P* = 0·002). No difference in cytokine levels between the two groups was found for TNF-α, IL-1β, IL-2, IL-4 and IL-8. After gluten-free diet, levels of IL-5, IL-12 and IL-10 decreased significantly (*P* < 0·001, *P* = 0·002 and *P* = 0·007) and IFN-γ levels were reduced (*P* = 0·059). Young children with coeliac disease detected by screening demonstrate elevated levels of serum cytokines at time of diagnosis. A prolonged systemic inflammation may, in turn, contribute to long-term complications known to be associated with untreated coeliac disease.

## Introduction

Cytokines are a heterogeneous group of signalling substances involved both in proinflammatory and anti-inflammatory actions and are believed to be an important part of the development of autoimmune disease 1. Coeliac disease arises in genetically susceptible individuals and is characterized by a T cell-driven inflammation in the proximal small bowel triggered by ingested gluten. Nearly all coeliac disease patients express autoantibodies against tissue transglutaminase (tTG), which is the marker for active disease 2.

Although cytokines have been mainly studied locally in the mucosal layer of the small intestine in coeliac disease, some studies have also examined systemic levels of cytokines 3,4, evaluated both as markers of disease activity as well as of disease remission after treatment with a gluten-free diet 5–8. In children with untreated coeliac disease, several studies have found increased levels of cytokines belonging to the T helper type 1 (Th1) pattern, and interferon (IFN)-γ in particular, compared with controls 4,5.

Circulating cytokines originating from an inflammatory disease in the gastrointestinal (GI) tract may result in extra-intestinal manifestations such as osteoporosis 9 and anaemia 10 or contribute potentially to lymphoma development 11. A systemically detected altered cytokine pattern could therefore be a link between coeliac disease and related complications as well as reflecting the effect of a gluten-free diet.

However, no study has assayed peripheral cytokines in children with asymptomatic coeliac disease in whom the phenotypical expression may differ from those with symptomatic coeliac disease. As many patients are expected to be clinically silent cases and at potential risk for long-term complications, due to a chronic systemic inflammation, more studies are warranted to explore if asymptomatic cases have signs of systemic inflammation reflected as altered levels of circulating cytokines.

The aim of the present study was to measure cytokines of Th1 and Th2 patterns in serum from young children with screening-detected coeliac disease at the time of diagnosis and after treatment with a gluten-free diet.

## Materials and methods

### Study population

Between 2000 and 2004, children born in the region of Scania in the southernmost part of Sweden were included in the Diabetes Prediction in Skane study (DiPiS) and Celiac disease Prediction in Skane study (CiPiS), which are two prospective cohort studies aiming at determining genetic, immunological and environmental factors for diabetes and coeliac disease. At birth, cord blood was collected for human leucocyte antigen (HLA) genotyping, and at 3 years of age the child was screened for coeliac disease with tissue transglutaminase (tTG) autoantibodies; cytokine measurements were performed on the same blood sample. Children were analysed for tTG autoantibodies and retested after 3 months if being initially tTG autoantibody-positive. Children persistently tTG autoantibody-positive at follow-up were defined as having coeliac disease autoimmunity (CDA) and referred to intestinal biopsy to confirm the diagnosis of coeliac disease. Children with a biopsy showing Marsh score 1 or greater were considered to have biopsy-proven coeliac disease and put on a gluten-free diet. Although not classified as coeliac disease according to the European Society for Pediatric Gastroenterology, Hepatology and Nutrition (ESPGHAN) criteria 12, children having a Marsh score 1 were included in the coeliac disease group because of HLA risk genotype, elevated tTG autoantibodies and histological signs of incipient coeliac disease 13–15. All children with CDA, regardless of biopsy result, were included in the cytokine measurements. In total, blood samples from 34 eligible children [mean 3·4 (range 3·2–4·1) years] were included for cytokine measurements, of whom 26 children had biopsy-confirmed coeliac disease [Marsh 1: three of 26 (11%) and Marsh 3: 23 of 26 (89%)] and eight children had normal intestinal biopsy (Marsh 0), albeit defined as having CDA. Serum samples from 68 tTG autoantibody-negative children matched for sample date, year of birth, gender and HLA genotype were selected as controls. From all children participating in the DiPiS study, including children put onto a gluten-free diet, a yearly blood sample was collected accessible for cytokine measurements.

The study was approved by the Regional Research Ethics Board at Lund University and one parent per child gave informed, written consent to participate in the CiPiS study.

### Transglutaminase autoantibody radioligand binding assay

Both immunoglobulin (Ig)A–tTG and IgG–tTG were measured with a radioligand binding assay, as described elsewhere 16. Briefly, human tTG was synthesized in the presence of 20 mCi 35S-methionine (Perkin Elmer LifeSciences, Inc., Boston, MA, USA) by *in-vitro* transcription and translation, as described previously. The IgA–tTG antigen/antibody complexes were isolated with 10% goat anti-human IgA agarose (Sigma, St Louis, MO, USA) and the IgG–tTG antigen/antibody complexes were separated with 30% protein A sepharose conjugate 4B (Zymed Laboratories Inc., San Francisco, CA, USA). Radioactivity [counts per minute (cpm)] was measured in a beta counter and the amount of tTG was expressed as units per millilitre (U/ml) computed from standard curves. Cut-off levels for a positive result were calculated using quantile–quantile plots from 398 healthy blood donors and set at 16 U/ml for IgA–tTG and 4 U/ml for IgG–tTG, respectively 16.

### Cytokine measurements

All serum samples were tested using an electrochemiluminescent multiplex sandwich enzyme-linked immunosorbent (Th1/Th2) assay (MesoScale, Gaithersburg, MD, USA), in which every cytokine was measured at the same time in one single sample (25 μl human serum). In the assay, the following cytokines and chemokine were measured: interferon (IFN)-γ, interleukin (IL)-10, IL-12p70, IL-13, IL-1β, IL-2, IL-4, IL-5, tumour necrosis factor (TNF)-α and IL-8, as per the manufacturer's protocol on a Sector 6000 instrument (http://www.mesoscale.com).

### Statistical methods

The cytokine levels were first examined in box-plots as continuous variables with log_10_ base transformation made to normalize the cytokine measurements. A Kruskal–Wallis one-way analysis of variance test tested for a significant overall shift in cytokine levels in cases and controls and the Mann–Whitney *U*-test examined identified sample pairs. Wilcoxon's signed-rank test tested for differences in distribution between two related samples. The median and interquartile range (IQR) for each cytokine in each group are used as descriptive measurements. The Bonferroni correction method was used for multiple comparisons. Statistical analysis was performed using spss version 21·0 (http://www-01.ibm.com/software/analytics/spss/). The figure was drawn using GraphPad PRISM (version 4). *P*-values less than 0·05 were considered statistically significant.

## Results

**Figure 1:**
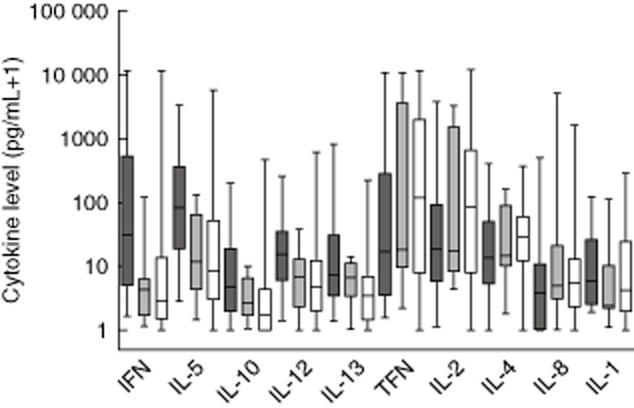
Box-and-whisker plot showing minimum and maximum value, 25% percentile, median and 75% percentile for cytokine levels on a log_10_ scale in children having biopsy-proven coeliac disease (*n* = 26, dark grey boxes), children having tissue transglutaminase antibodies and normal biopsy (*n* = 8, grey boxes) and controls (*n* = 68, white boxes).

Levels of each of the assessed serum cytokines for children with coeliac disease, CDA and matched controls are given in Fig. 1. There was an overall shift in cytokine levels in five of 10 serum cytokines between the three groups (Fig. 1). No difference was detected when comparing children having CDA with controls for any of the cytokines in the analysis; consequently, CDA and their matched controls were excluded in further analysis.

When comparing children with coeliac disease and matched controls, Th1 serum cytokine levels for IFN-γ and IL-12p70 were higher in children with coeliac disease compared to controls (Table 1). Moreover, the Th2-associated cytokines IL-5, IL-10 and IL-13 were elevated in children with coeliac disease compared to controls (Table 1). The difference between the groups persisted after correcting for multiple comparisons. The result also remained unchanged if analysing subgroups of children having Marsh scores 1 and 3, respectively (not shown). No difference was found between children with coeliac disease and controls for TNF-α, IL-2, IL-1β, IL-4 and IL-8 (Table 1).

**Table 1:** Median and interquartile range of cytokine levels among children having biopsy-proven coeliac disease and controls (negative for tissue transglutaminase antibodies)

Cytokine	Coeliac disease (*n* = 26)	Controls (*n* = 52)	*P*-value^‡^
(pg/ml)	Median (IQR†)		
IFN-γ	29·8 (4·1–492·4)	1·7 (0·5–13·3)	<0·001
IL-5	81·7 (18·1–339·1)	7·1 (1·3–50·7)	<0·001
IL-10	3·7 (1·1–17·0)	0·8 (0–3·7)	0·001
IL-12p70	14·1 (5·7–32·0)	3·7 (0·9–9·7)	0·001
IL-13	6·3 (2·6–27·4)	2·5 (0·04–5·7)	0·002
TNF-α	15·8 (2·6–260·2)	95·0 (6·0–2033·2)	0·100
IL-2	17·9 (4·9–88·0)	67·3 (6·7–421·8)	0·132
IL-1β	5·0 (1·7–25·1)	2·7 (0·8–17·7)	0·135
IL-4	12·5 (4·4–47·8)	26·9 (7·9–55·1)	0·185
IL-8	2·8 (0·1–9·3)	4·0 (1·3–11·0)	0·188

† Interquartile range.

‡ Mann–Whitney *U*-test for differences in distribution between two independent samples. IL = interleukin; IFN = interferon; TNF = tumour necrosis factor.

Serum samples were collected in 19 of 26 children with coeliac disease after starting a gluten-free diet after 1·5 ± 0·36 years [median ± standard deviation (s.d.)] and analyses performed for cytokines IFN-γ, IL-5, IL-10, IL-12p70 and IL-13 (Table 2). IL-5, IL-10 and IL-12p70 showed a significant reduction in cytokine levels after starting a gluten-free diet and IFN-γ levels were reduced, although not significantly (Table 2). No difference was found for IL-13 at diagnosis compared to after a gluten-free diet.

**Table 2:** Median and interquartile range of cytokine levels among children having biopsy-proven coeliac disease at time of diagnosis and after start of a gluten-free diet

Cytokine	Level at diagnosis (*n* = 19)	Level after a GFD†	*P*-value§
(pg/ml)	Median (IQR‡)		
IFN-γ	14·8 (3·2–451·4)	7·8 (4·7–14·0)	0·059
IL-5	38·4 (15·3–127·1)	0·19 (0·10–0·34)	<0·001
IL-10	2·0 (0·86–15·7)	0·74 (0·20–1·8)	0·007
IL-12p70	12·3 (2·4–34·0)	0·39 (0·19–0·71)	0·002
IL-13	5·1 (2·1–18·4)	5·7 (2·7–17·9)	0·841

† Duration of time on a gluten-free diet (GFD) was 1·5 ± 0·36 years (median ± standard deviation).

‡ Interquartile range.

§ Wilcoxon's signed-rank test for differences in distribution between two related samples. IL = interleukin; IFN = interferon.

## Discussion

In this study, cytokines belonging both to the Th1 and Th2 patterns were assayed in serum of young children screened for coeliac disease and in controls matched for sample date, year of birth, gender and HLA. Several cytokines were elevated significantly in children with screening-detected coeliac disease compared to controls, suggesting that there is also a systemically detectable immunological response in small children with no overt clinical signs of chronic disease. Our results show that children with untreated coeliac disease, albeit detected by screening, show signs of a systemic inflammation reflected as an increased response of proinflammatory cytokines in serum blood samples at the time of diagnosis. This finding is of special interest to physicians managing children with coeliac disease, and indicates that untreated individuals are at risk for long-term complications due to a systemic inflammation regardless of being asymptomatic and detected by screening.

Th1-derived cytokines are considered to be associated with autoimmune conditions 1, and former studies of coeliac disease have characterized the immune response as typical Th1 4,17. In this study, both levels of IFN-γ and IL-12 from the Th1 pattern were elevated in young children with coeliac disease compared to controls. This confirms previous studies of detecting high levels of IFN-γ both locally in the intestinal mucosa 18 as well as in serum from children with coeliac disease 17. Thus, screening-detected coeliac disease in young children seems to have a typical IFN-γ response detected in serum characteristic of coeliac disease. Conversely, elevated levels of IL-12, considered to be an inducer of IFN-γ in intestinal inflammation 19, have not been associated with coeliac disease in other studies, although studied only in cells originating from intestinal biopsies 20,21. We found no difference between cases and controls concerning TNF-α, IL-2, IL-1β and the chemokine IL-8. TNF-α has formerly been found to be both elevated in coeliac disease compared to controls 3 as well as unchanged 6. In our study, the median value of TNF-α was lower in coeliac disease compared to controls, which has been shown in a former study of cytokine production in intestinal T cells in coeliac disease 22. In the same study of cytokines of mucosal T cells, production of IL-2 was found to be depressed compared to controls and similar to the findings of our study. Thus, it seems that screening-detected coeliac disease is characterized both by elevated as well as down-regulated cytokine levels in serum within the Th1 cytokine group.

Our finding of cytokines IL-5, IL-10 and IL-13 belonging to the Th2 response being elevated significantly in coeliac disease was surprising, as a Th2 cytokine pattern has not been considered previously to be central in the immune reaction in coeliac disease. IL-5, involved in eosinophilic differentiation, has not been studied previously in serum of coeliac disease patients, but has been detected in eosinophilic cells from intestinal mucosa of coeliac disease patients 23. The anti-inflammatory cytokine IL-10, known as an important immunomodulator of the intestinal tract 24, has in former studies been both up-regulated 3,25 as well as unchanged 4,5 in coeliac disease patients compared to controls. IL-13, but not IL-10, was found elevated in a study of refractory coeliac disease 26. IL-13 of the GI tract is associated mainly with ulcerative colitis and eosinophilic oesophagitis 27, and the finding in our study of elevated levels of IL-13 in serum of children with screening-detected coeliac disease may shed new light on the role and function of this particular cytokine. Conversely, the related Th2 cytokine IL-4 was low in coeliac disease compared to controls (although not significantly), similar to other studies 4,5, reflecting the inverse relationship between IFN-γ and IL-4 production 28. Thus, the cytokine response in children with screening-detected coeliac disease shown in this study should be categorized as a combination of Th1 and Th2 patterns.

A drawback of this study is that we did not measure cytokine expression in the mucosa, which does not enable us to compare serum cytokine levels and mucosal cytokine expression. Former studies have shown diverging results concerning the possibility of cytokines in peripheral blood reflecting mucosal immune responses 18,29. As this study was based on screening-detected coeliac disease, i.e. asymptomatic children potentially at risk for coeliac disease-related extra intestinal complications, cytokines detected in peripheral blood were considered to be the more relevant part of the immune response to measure 30.

Different cytokine patterns could potentially reflect various clinical manifestations and phenotypes, as illustrated in patients with dermatitis herpetiformis with and without coeliac disease 31. In this study, we did not measure cytokine patterns in clinically detected coeliac disease or grade the clinical manifestation among the screening-detected cases, making such conclusions impossible. However, a former study found no correlation between clinical presentation and levels of serum cytokines 3.

Several studies of clinical coeliac disease have revealed alterations in cytokine levels after starting a gluten-free diet 3,4,6,7 compared to time of diagnosis, but also that some cytokines detected in serum continue to be elevated compared to controls after 1 year of treatment 6. Therefore, it is also of great importance to study the anti-inflammatory effect of a gluten-free diet on the cytokine pattern in children with screening-detected coeliac disease. In our study, IFN-γ, IL-5, IL-10 and IL-12p70 levels were reduced after treatment with a gluten-free diet, which has been confirmed in former studies 3,5,32. Coeliac disease-related complications associated with these cytokines could therefore benefit from a gluten-free diet. IL-13 levels, however, were not altered after starting a gluten-free diet, indicating that IL-13 is not related directly to gluten ingestion.

Measurements of cytokines are known to be affected by storage time and number of thaw cycles 33. As the samples from this study were collected at different time-points between diagnosis and after a gluten-free diet, there is a potential risk that the decrease in cytokine levels between diagnosis and after a gluten-free diet may have been affected over time. However, all measurements were performed on stored frozen samples prior to analysis and thawed for the purposes of this study only, to keep to a minimum any difference in usage of samples that may have changed the validity of our results.

The diagnosis of coeliac disease was based on the ESPGHAN criteria 12, but children with repeatedly elevated tTG antibody levels, HLA risk genotype and duodenal biopsy classified as Marsh 1 were also included. Individuals with mild histological alterations have been argued as not having coeliac disease 34,35, but some studies indicate that individuals with coeliac disease and mild enteropathy also experience classical symptoms, alterations in laboratory findings and associated conditions and could benefit from a gluten-free diet 13–15. In our material only three of 26 (11%) of the children had a Marsh score <3, thus representing only a minor part of the coeliac disease group. The result remained unchanged when analysing both subgroups (Marsh 1 and Marsh 3), indicating that screening-detected persistently tTG autoantibody-positive children having only mild histological alterations have the same systemic signs of mucosal inflammation as children with classic coeliac enteropathy.

In conclusion, signs of a systemic inflammatory response reflected as elevated levels of serum cytokines were found in 3-year-old children with screening-detected coeliac disease at the time of diagnosis. A chronic systemic inflammation due to unrecognized disease in young children may contribute to an increased risk for other systemic inflammatory disorders and long-term complications associated with prolonged untreated coeliac disease, which should be accounted for when screening is performed in young children.
